# Influence of hypertension diagnosis and subjective life expectancy on health behaviors among middle-aged and older Chinese adults

**DOI:** 10.3389/fpubh.2024.1489284

**Published:** 2025-01-06

**Authors:** Xiaoxiao Liu, Xiaoyi Ji, Honghong Xia, Yuxin Tian, Weihong Zheng, Linyun Fu, Peiyuan Qiu, Yang Wan

**Affiliations:** ^1^Department of Neurology, West China School of Public Health and West China Fourth Hospital, Sichuan University, Chengdu, Sichuan, China; ^2^West China School of Public Health and West China Fourth Hospital, Sichuan University, Chengdu, Sichuan, China; ^3^Crown Family School of Social Work, Policy, and Practice, University of Chicago, Chicago, IL, United States

**Keywords:** hypertension awareness, health behavior modification, subjective survival expectations, China, subjective life expectancy (SLE)

## Abstract

**Objectives:**

Hypertension can lead to significant health complications if left unmanaged due to unhealthy behaviors. This study investigates hypertension related health behaviors of middle-aged and older Chinese adults, investigating whether a hypertension diagnosis and individuals’ subjective life expectancy (SLE) might prompt positive changes in their health behaviors.

**Methods:**

The participants in this study were Chinese adults aged 45 years and older, selected from the 2013–2020 China Health and Retirement Longitudinal Study. Linear mixed-effects models were employed to investigate the influence of receiving a hypertension diagnosis, as well as SLE, on hypertension related behaviors.

**Results:**

Among the respondents, 27.65% reported doctor-diagnosed hypertension, while 19.91% of those who were undiagnosed with hypertension had measured hypertension at baseline. Of those diagnosed with hypertension, only 46.97% in 2013 had their blood pressure within the normal range in 2013, and this slightly improved to 47.80% in 2015. Both receiving a hypertension diagnosis (β = 0.41, 95% CI: 0.37–0.43) and having a low-SLE (β = 0.06, 95% CI: 0.03–0.09) were associated with healthier behavior. Interestingly, individuals with measured hypertension exhibited the highest SLE but the lowest health behavior scores.

**Discussion:**

Although individuals diagnosed with hypertension are able to recognize the dangers of the condition and take proactive steps to improve their health, high blood pressure remains uncontrolled in almost half of them. Those with measured hypertension often lack awareness of hypertension and unhealthier behaviors. Therefore, there is a critical need to enhance hypertension awareness and promote healthier behaviors among both diagnosed individuals with uncontrolled blood pressure and those unaware of their hypertension.

## Introduction

Hypertension has become a prevalent issue for the majority of middle-aged and older adults in China, with the prevalence increasing over the years (1, 2). It is a significant risk factor for disability-adjusted life years (DALYs) worldwide, contributing to 10.4 million deaths and 218 million DALYs (3). Hypertension increases the mortality from cardiovascular diseases such as stroke and ischemic heart disease in older adults (4, 5). It may also lead to psychological problems (6). Studies have confirmed that receiving treatment can reduce the burden of disease in people with hypertension, and there is no significant difference in the mortality between patients with well-controlled blood pressure and those with normotension (7, 8). However, in China, awareness, management, and control of hypertension are still generally insufficient, and only one-quarter of the patients have received treatment (9). Therefore, it is clear that awareness and management of hypertension should be promoted among the Chinese population.

There are several factors contributing to the mismanagement of blood pressure in patients. Firstly, hypertension is often asymptomatic, and if patients are not diagnosed, they may remain unaware of the condition and therefore not engage in treatment or behavioral management. Secondly, even among diagnosed patients, some may struggle to adhere to healthy behaviors, leading to further mismanagement.

The Common Sense Model of Self-Regulation (CSM) proposes that patients’ response to a health threat depends on their awareness of the threat, perceptions of the threat and potential therapeutic behaviors, which then guide their action plans (10, 11). Patients’ motivation to take control measures is often driven by their perception of the disease as a life-threatening condition. Subjective life expectancy (SLE), often used to gauge an individual’s expectation of reaching their life expectancy (12–14, 28). If someone perceives themselves to be at a higher risk of health issues, they may anticipate a decrease in life expectancy, leading them to adopt positive health behaviors in order to improve their chances of survival in the future. While SLE has been useful for health investment planning (15) and care planning (16), there is limited research exploring the impact of SLE on health behaviors. In this study, we hypothesize that individuals diagnosed with hypertension would have a lower SLE, which would, in turn, promote healthier behavior patterns.

To test this hypothesis, our analysis is divided into three parts. First, we describe the distributions of hypertension, SLE, and health behaviors among respondents from 2013 to 2018. Second, we investigate hypertension status of respondents in 2013 and 2015. Additionally, we examined relationship between different hypertension status and SLE and health behaviors. Third, we investigate whether a Low-SLE is associated with an increase in healthier behaviors. We do this by evaluating the relationship between SLE and health behaviors, while adjusting for the diagnosed status of hypertension. Furthermore, we analyzed the relationship between SLE and health behaviors among individuals diagnosed with hypertension. To ensure the robustness of our findings, we conduct additional analyses with alternate specifications that include individual fixed effects.

## Materials and methods

### Data

Data are from the China Health and Retirement Longitudinal Study (CHARLS), a national longitudinal survey initiated in 2011. This survey is conducted every two to 3 years and involves a sample of approximately 10,000 households from 150 counties. The survey includes 17,000 respondents aged 45 and above. Further information regarding the design of CHARLS can be found in the relevant literature (17).

All participants or their legal representatives provided written informed consent. The Biomedical Ethics Committee of Peking University (IRB00001052-11015) provided ethical approval for the study.

**Figure 1:**
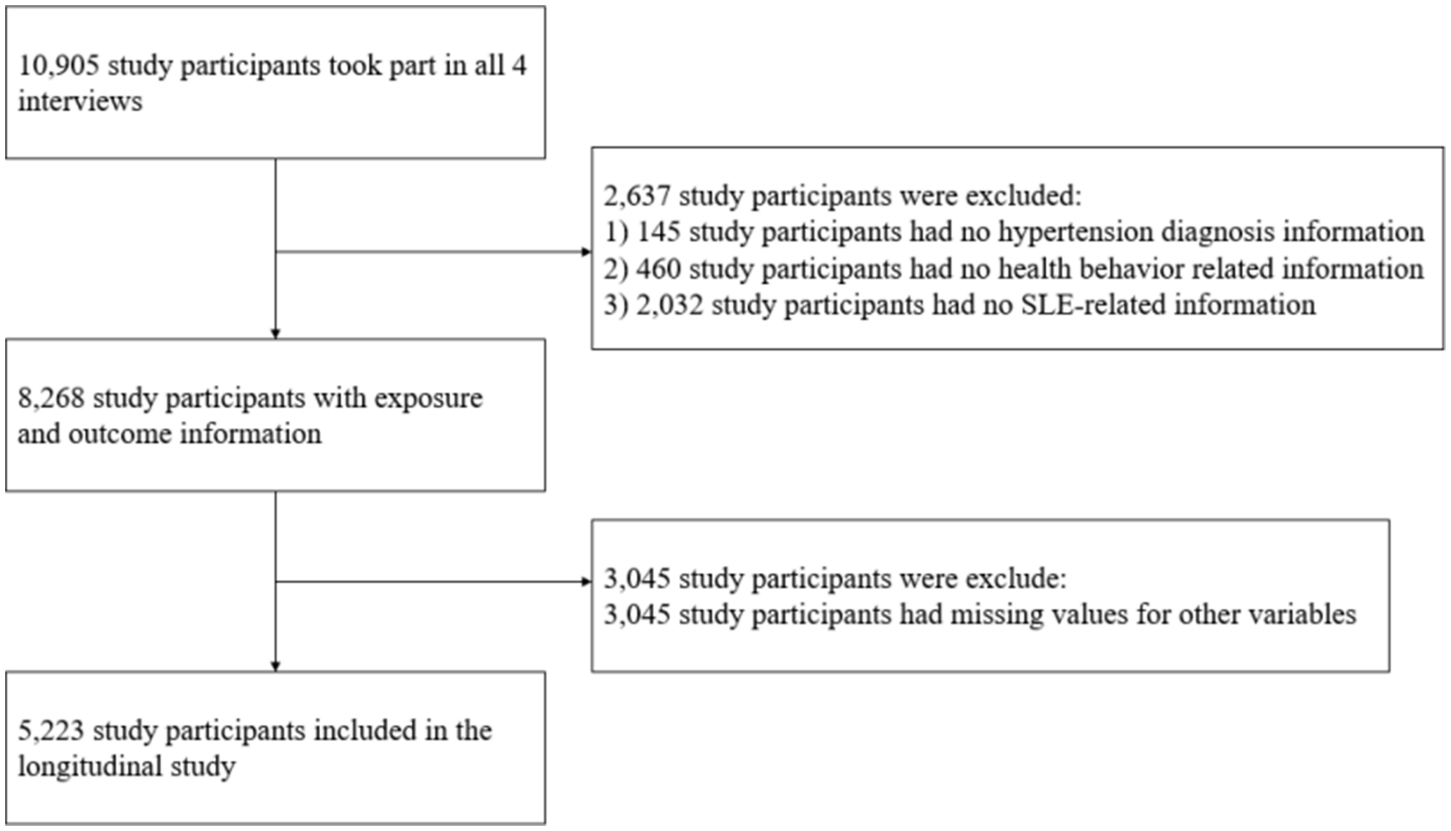
Flowchart of sample recruitment.

Figure 1 provides an overview of the participant selection process in this investigation. Wave 2 (2013) of the CHARLS survey was designated as the baselines for this study. The inclusion criteria encompassed: (1) participants aged 45 years and above; and (2) individuals who participated in all four waves of the survey (2013, 2015, 2018 and 2020). Exclusion criteria involved participants with missing data on variables used in the analysis. As a result, the final sample contained of 5,223 individuals.

### Measure

#### Diagnosed and measured hypertension

All participants were require to indicate if they had been diagnosed with hypertension by doctors. If a participant answered “yes” to this question, they were labeled as diagnosed hypertension.

Blood pressure measurements were taken during the 2013 and 2015 waves. Each subject’s systolic and diastolic blood pressures were measured three times by one investigator using an HEM-7112 electronic monitor (Omron, Kyoto, Japan). The mean blood pressure value was then calculated for each subject. According to the criteria set by the World Health Organization (18), individuals who had a mean systolic blood pressure of 140 mm Hg or higher and/or a mean diastolic blood pressure of 90 mm Hg or higher, but did not report a previous diagnosis of hypertension, were classified as having measured hypertension.

#### Subjective life expectancy (SLE)

SLE in the CHARLS was determined using the following question “What is the chance that you will live to be [75 (if respondent’s current age is 64 or less)/80 (if age is between 65 and 69)/85 (if age is between 70 and 74)/90 (if age is between 75 and 79)/95 (if age is between 80 and 84)/100 (if age is between 85 and 89)/105 (if age is between 90 and 94)/110 (if age is between 95 and 99)/115 (if age is 100 or more)]?.” Participants provided their response on a 5-point Likert scale, ranging from 1 (almost impossible) to 5 (almost certain). Based on the distribution of SLE scores, they were categorized as follows: scores of 1–2 were classified as Low-SLE, a score of 3 as Middle-SLE, and scores of 4–5 as High-SLE (Supplementary Figure S1).

#### Health behaviors

##### Tobacco avoidance

To determine smoking status, two questions were utilized: “Have you ever chewed tobacco, smoked a pipe, smoked self-rolled cigarettes, or smoked cigarettes/cigars?” and “Do you still have the habit, or have you totally quit?.” Respondents who indicated that they had never smoked or had quit were classified as non-smokers, assigned a score of 1; while those who reported they were still smoking were classified as smokers, assigned a score of 0.

##### Alcohol avoidance

To determine drinking status, participants were asked the question, “Did you consume any alcoholic beverages, such as beer, wine, or liquor in the past year? “. Those who reported drinking in the last year were classified as drinkers and scored as 0. Conversely, those who reported never drinking in the past year were classified as non-drinkers and scored as 1.

##### Physical activity

Physical activity levels are categorized into three intensities: (1) Vigorous Physical Activity (VPA): This category includes activities that significantly increase breathing difficulty, such as heavy lifting, digging, ploughing, aerobics, fast bicycling, and cycling with a heavy load, as classified in CHARLS. (2) Moderate Physical Activity (MPA): This category includes activities that moderately increase breathing difficulty, such as carrying light objects, bicycling at a regular pace, or mopping the floor, as classified in CHARLS. (3) Low Physical Activity (LPA): This category includes walking activities that individuals engage in for recreation, sport, exercise, or leisure, as classified in CHARLS.

In our study, physical activity was defined as engaging in VPA for at least 30 min, more than once a week with; engaging in MPA for at least 30 min, more than three times a week; or engaging in LPA for at least 30 min, more than five times a week. Respondents who met this standard were assigned a score of 1, while those who did not meet the standard received a score of 0.

##### Medication use

To assess medication use for hypertension control among individuals diagnosed with hypertension, they were asked the question, “Are you currently taking any medications, including both traditional Chinese medicine and Western modern medicine to treat or control your hypertension? “Based on their response, respondents were assign a score of 1 if they reported taking any type of medication for hypertension control, and a score of 0 if they reported not taking any medication.

#### Blood pressure monitoring

For determining the frequency of respondents’ self-monitoring blood pressure check, we asked the question, “During the last 12 months, how many times have you had your blood pressure examined? “Responses were categorized into four groups: 0 for no checks, 1 for one to six checks, 2 for seven to twelve checks, and 3 for more than 12 checks. The total score for this behavior ranged from 0 to 3.

In our study, we examined general health behaviors in all respondents. For individuals with hypertension, we further investigated their general health behaviors as well as their hypertension-control related health behaviors, collectively referred to as hypertension health behavior. General health behavior included tobacco avoidance, alcohol avoidance and regular engagement in physical activity. The total score for general health behavior was calculated by summing the scores for the three mentioned behaviors, ranging from 0 to 3. Hypertension-control related health behaviors encompassed consistent medication use for hypertension and regular monitoring of blood pressure. The score for hypertension-control related health behaviors was calculated by summing the scores for consistent medication use and regular monitoring of blood pressure, ranging from 0 to 4. Among individuals with diagnosed hypertension, we further calculated the hypertension health behavior score. This score was defined as the sum of general health behaviors and hypertension-control related health behaviors, ranging from 0 to 7.

#### Covariates

We included several covariates in our study that may confound association between SLE and health behaviors. These covariates encompassed demographic, socio-economic and health-related characteristics.

The demographic variables included age, gender, and marital status. Age was treated as a continuous variable, while gender was coded as a binary variable (0 for female, 1 for male). Marital status was also coded as a binary variable (1 for married, 2 for unmarried).

The socioeconomic variables comprised the location of the current residence, education, and pension. The location of the current residence was coded as a binary variable (0 for urban, 1 for rural). Education was categorized into three levels: 1 for illiterate, 2 for elementary school and below, and 3 for middle school and above. Pension was coded as a binary variable, with 1 indicating the receipt of any pension income in the past year and 0 indicating no pension income received.

Additionally, we included health-related variables in our analysis. The degree of disability was assessed based on three activities in which individuals may require assistance: bathing, dressing, and eating. We assigned a value of 0 to indicate no assistance needed and a value of 1 to indicate assistance needed for each activity. The degree of disability was then calculated as the sum of assistance needs, ranging from 0 to 3.

### Statistical analysis

Descriptive analyses were performed to investigate the distribution of hypertension, self-reported life expectancy, health behavior scores, and other participant characteristics from 2013 to 2020. Furthermore, we described the proportion of respondents with measured hypertension in Table 1.

**Table 1:** Distribution of characteristics of participants across 2013 to 2018 (*N* = 5223).

	2013		2015		2018		2020	
	Mean/*n*	SD/%	Mean/*n*	SD/%	Mean/*n*	SD/%	Mean/*n*	SD/%
Gender								
Male	2674	51.20						
Female	2549	48.80						
Age, mean	58.11	8.06	60.10	8.06	63.10	8.06	65.11	8.06
Education								
Illiterate	1024	19.61						
Elementary school and below	2068	39.59						
Middle school and above	2131	40.80						
Marital status								
Married	4753	91.00	4500	86.16	4118	78.84	3930	75.24
Unmarried	470	9.00	723	13.84	1105	21.16	1293	24.76
Residence								
Urban	1805	34.56						
Rural	3418	65.44						
ADLWA, mean	0.09	0.39	0.12	0.46	0.15	0.50	0.18	0.53
Pension								
No	1827	34.98						
Yes	3396	65.02						
SLE								
Low-SLE	1618	30.98	1754	33.58	1841	35.25	1802	34.50
Middle-SLE	1658	31.74	1874	35.88	1901	36.39	1996	38.22
High-SLE	1947	37.38	1595	30.54	1481	28.36	1425	27.28
Diagnosed hypertension								
Diagnosed hypertension	1444	27.65	1723	32.99	2198	42.08	2526	48.36
No diagnosed hypertension	3779	72.35	3500	67.01	3025	57.92	2697	51.64
General health behavior score, mean	1.39	0.88	1.55	0.86	1.81	0.90	2.26	1.05
Hypertension health behavior score among diagnosed patients^a^, mean	3.57	1.67	3.79	1.67	3.82	1.53	3.91	1.54

ADLWA = Activities of daily living by Wallace; SLE = Subjective life expectancy.

^a^ Evaluated for the subset of the analytic sample who had diagnosed hypertension and were surveyed in 2013–2020.

Participants were categorized into different blood pressure statuses, including those with no hypertension, measured hypertension, diagnosed hypertension with normal blood pressure, and diagnosed hypertension with abnormal blood pressure. To compare the differences in SLE and general health behaviors scores among the four groups, we used the Kruskal-Wallis rank-sum test. Subsequently, pairwise comparisons between each pair of groups were conducted using Dunn’s test. To examine the difference in hypertension health behaviors between individuals with diagnosed hypertension with normal and abnormal blood pressure, we employed the Wilcoxon rank-sum test.

To explore the impact of SLE on both general health behaviors and hypertension health behavior, linear mixed effects models were employed. These models incorporated survey time as the first level and individuals as the second level. These models adjusted several covariates, including demographic variables, socio-economic variables and health-related variables.

To ensure the robustness of the models, fixed-effects models were also conducted to account for potential changes in variables that could influence the model outcomes. All statistical analyses in this study were performed using R 4.2.2. A *p*-value of less than 0.05 was considered statistically significant.

## Results

### Descriptive statistics

Table 1 displays the distribution of characteristics of the respondents for the four waves of surveys conducted in 2013, 2015, 2018 and 2020. It is notable that the number of individuals with hypertension has exhibited an upward trend over time. In 2013, 27.65% of the respondents were diagnosed with hypertension, whereas in 2020, this percentage had increased to 48.36%. Simultaneously, there has been a gradual rise in the adoption of healthier behaviors. The SLE of the respondents has shown a decline. The proportion of respondents with high SLE at baseline was 37.38%. However, by 2020, this percentage had decreased to 27.28%.

**Figure 2:**
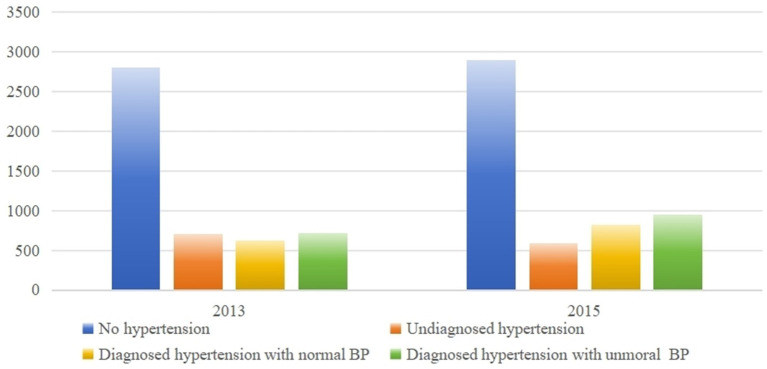
Descriptive statistics of respondents’ blood pressure status.

Using data from the 2013 and 2015 waves, we analyzed respondents’ hypertension status during those years. It is important to note that data from 2018 and 2020 were not included in this specific analysis as the surveys conducted in that year did not include the blood pressure measurement program. In 2013, we observed that 19.91% (658 out of 3305) of respondents who reported being undiagnosed with hypertension had measured hypertension. Similarly, in 2015, this percentage remained high at 16.94% (514 out of 3034). Furthermore, less than half of individuals diagnosed with hypertension had their hypertension under control, with only 46.97% in 2013 and 47.80% in 2015 having their blood pressure within the normal range (Figure 2).

### Distributions of health behaviors and SLE among older adults with different blood pressure status

We conducted a comparison of health behavior scores and SLE among respondents with different blood pressure statuses, including those with no hypertension, measured hypertension, diagnosed hypertension with normal blood pressure, and diagnosed hypertension with abnormal blood pressure. As shown in Table 2, significant differences were observed in both health behavior scores and SLE across the four groups.

**Table 2:** The difference of health behavior and SLE between four group respondents in 2013 and 2015 (*N* = 4559).

	No hypertension	Measured hypertension	Diagnosed hypertension with normal BP	Diagnosed hypertension with unmoral BP
hypertension health behavior score, mean	(*p* < 0.001)			
	–	–	3.71	3.48
General health behavior score, mean	(*P* < 0.001)			
	1.39	1.32	1.46	1.47
SLE	(*P* < 0.001)			
Low-SLE	30.42	25.78	37.22	40.85
Middle-SLE	33.06	33.45	29.13	26.84
High-SLE	36.52	40.77	33.65	32.31

SLE = Subjective life expectancy.

Supplementary Table 1 provides further details on these differences between the groups. Specifically, respondents with measured hypertension exhibited the largest proportion of High-SLE (44.84%), indicating a higher expectancy, and the lowest score of general health behaviors.

### Contributions of SLE to general health behavior

After adjusting for covariates, the results of the linear mixed-effects model in Table 3 examining the impact of being diagnosed with hypertension on general health behaviors reveal a significant positive correlation with the score of general health behaviors (β = 0.41, 95% CI: 0.37–0.43). Furthermore, controlling for covairates and hypertension status, low-SLE was found to be negatively correlated with the score of general health behaviors (β = 0.06, 95% CI: 0.03–0.09). It serves as a motivating factor for individuals to adopt healthier behaviors. These findings highlight the importance of considering both SLE and hypertension diagnosis when promoting and encouraging healthy behaviors among middle-aged and older adults.

**Table 3:** Linear mixed-effects model of SLE on general health behavior.

	Model 1	Model 2
	β (95% CI)	β (95% CI)
Hypertension diagnosis (Ref: No hypertension diagnosis)		
Hypertension diagnosis	0.41 (0.37, 0.43)***	0.41 (0.37, 0.43)***
SLE (Ref: High-SLE)		
Middle-SLE	–	0.05 (0.02, 0.09)***
Low-SLE	–	0.06 (0.03, 0.09)***
Age		
	0.01 (0.00, 0.01)***	0.01 (0.00, 0.01)***
Gender (Ref: Male)		
Female	0.96 (0.93, 0.99)***	0.96 (0.93, 0.98)***
Marital status (Ref: Married)		
Unmarried	−0.01 (−0.04, 0.03)	−0.01 (−0.05, 0.02)
Education (Ref: Illiterate)		
Elementary school and below	0.01 (−0.03, 0.03)	0.01 (−0.03, 0.03)
Middle school and above	0.02 (−0.02, 0.05)	0.02 (−0.02, 0.06)
Residence (Ref: Urban)		
Rural	−0.08 (−0.11, −0.06)***	−0.08 (−0.12, −0.06)***
ADLWA		
	0.02 (−0.01, 0.05)	0.01 (−0.01, 0.04)
Pension (Ref: No)		
Yes	0.01 (−0.04, 0.03)	0.00 (−0.04, 0.04)
Constant		
	0.83 (0.25, 1.43)***	0.82 (0.23, 1.40)***
N observations	5, 223	5, 223
R-squared	0.43858	0.4387128
AIC	42617.92	42614.38
BIC	42711.67	42715.94

CI = Confidence interval; ADLWA = Ability of daily living by Wallace; SLE = Subjective life expectancy; ***p < 0.001; **p < 0.01; *p < 0.05.

### Impact of SLE on hypertension health behavior in participants with diagnosed hypertension

Table 4 demonstrates the factors that contribute to improvements in health behaviors among people with hypertension. After controlling for covariant variables, the analysis revealed a significant negative correlation between SLE and the score of health behaviors compared to those with the highest SLE (β = 0.06, 95% CI: 0.01–0.10; β = 0.07, 95% CI: 0.02–0.11).

**Table 4:** Linear mixed-effects model of SLE on hypertension health behavior among individuals diagnosed with hypertension.

	Model 3
	β (95% *CI*)
SLE (Ref: High-SLE)	
Middle-SLE	0.06 (0.01, 0.10)*
Low-SLE	0.07 (0.02, 0.11)**
Age	
	0.01 (1.00, 0.01)***
Gender (Ref: Male)	
Female	0.93 (0.89, 0.96)***
Marital status (Ref: Married)	
Unmarried	−0.06 (−0.11, −0.01)*
Education (Ref: Illiterate)	
Elementary school and below	−0.03 (−0.07, 0.02)
Middle school and above	−0.01 (−0.06, 0.04)
Residence (Ref: Urban)	
Rural	−0.15 (−0.19, −0.12)***
ADLWA	
	0.00 (−0.03, 0.03)
Pension (Ref: No)	
Yes	−0.03 (−0.08, 0.02)
Constant	
	1.22 (0.19, 2.25)***
N observations	2, 496
R-squared	0.1696214
AIC	28575.97
BIC	28659.02

CI = Confidence interval; ADLWA = Ability of daily living by Wallace; SLE = Subjective life expectancy; ***p < 0.001; **p < 0.01; *p < 0.05.

### Robustness analysis

To address potential instability in the observations due to changes in SLE values and health behavior scores at different time points, we conducted a robustness analysis. A total of 1,051 respondents experienced changes in their SLE values, while 3,152 respondents had changes in their health behavior scores. To control for differences in individual characteristics, fixed-effects models were fitted to Models 1 and 2 as well as model 3. The results of the robustness analysis, presented in supplementary Tables 2–4, indicate that changes in the observations did not have a significant impact on the results. The fixed-effects models confirm that diagnosed hypertension motivates respondents to adopt healthy behaviors, and that lower SLE is associated with a higher health behavior score, both in the general population and among people with hypertension. These findings provide additional support for the robustness of our main results.

## Discussion

In this study, we observed that middle-aged and older adults diagnosed with hypertension had lower SLE compared to those who were not diagnosed with hypertension, including those with no hypertension and those with measured hypertension. This finding aligns with previous research indicating that being diagnosed with hypertension affects patients’ perceptions of their future survival probability (19). Additionally, we found that individuals with diagnosed hypertension exhibited better health behaviors than those with undiagnosed hypertension, including individuals without hypertension and those with measured hypertension. This finding is consistent with previous studies that have emphasized the importance of accurate diagnosis and awareness of hypertension (20).

Moreover, we observed a significant association between Low-SLE and better health behaviors. This suggests that concerns about future life expectancy motivate individuals to undertake proactive health actions. This phenomenon may be attributed to individuals with hypertension recognizing the importance of controlling their blood pressure through specific behaviors like medication adherence. These findings are consistent with CSM model, which suggests that patients take coping measures due to the perceived life-threatening effects of their disease condition and strive to change their health status (11).

Notably, individuals whose hypertension was under control had a higher proportion of individuals indicating low SLE compared to those with no hypertension. Despite their hypertension being under control, they self-perceived themselves as less healthy compared to those with no hypertension (19, 21), and even lower than those with measured hypertension. It can be assumed that the reason for their pessimism about their future lives was not the high blood pressure itself, but rather the fact that they were diagnosed as hypertension. On one hand, SLE can serve as a motivator for adopting healthier behavior, as individuals may take proactive measures to counteract the potentially life-threatening effects of hypertension. However, it is important to consider that studies have also shown that a better SLE correlates with better overall health outcomes (22) since it could promote mental health (23). Given that controlled hypertension is not life-threatening, it is important to address and potentially change older adults’ perception of their health status. This suggests that assisting individuals diagnosed with hypertension in developing an accurate perception of their health condition can facilitate the promotion of improved health outcomes.

Furthermore, one group of older adults that requires our attention is those who had measured high blood pressure but did not have diagnosed hypertension. We found that 14.5 and 11.3% older adults in 2013 and 2015, respectively, fell into this category. These individuals had the highest subjective life expectancy but exhibited the worst health behaviors. These findings suggest that individuals with measured hypertension may have a sense of well-being and may be less motivated to change unhealthy behaviors. Their unawareness or refusal to acknowledge their hypertension status puts them at high risk for uncontrolled hypertension (24) and hypertension related diseases, such as cardiovascular (25) and cerebrovascular disease (26), and potentially generates significant health burden in the future (27). Therefore, it is important to address this issue and provide support and education to encourage healthier behaviors among individuals with measured hypertension, as maintaining healthy habits is crucial for long-term health outcomes.

### Limitations and future directions

It is important to acknowledge several limitations of this study. Firstly, despite utilizing a longitudinal dataset, it is crucial to recognize that as an observational study, definitive causal relationships cannot be established. Therefore, the possible of reverse causality cannot be completely eliminated. Second, our understanding of participants’ blood pressure is constrained by the reliance on average values obtained from three measurements taken during a single interview, potentially introducing measurement bias. Third, there is a lack of information regarding why individuals may fail to recognize their hypertension status. Considering the positive impact of raising awareness about hypertension and its associated risks on fostering healthier behaviors, future research should explore barriers hindering individuals from acknowledge their disease status. Additionally, our understanding of patients’ medication usage relies on their response to a single survey question, which only asks whether they are taking medication without specifying the names of the medications. This may not accurately reflect whether patients are taking the correct antihypertensive medications. Future research should assess medication usage in more detail to obtain more accurate results.

Furthermore, considering that a significant proportion of individuals diagnosed with hypertension fail achieve blood pressure control, despite the availability of evidence-based interventions, a shift toward implementation science is warranted. Through rigorous implementation science methodologies, future research is expected to bridge the gap between knowledge and action, ultimately improving hypertension management and reducing associated health risks.

## Conclusion

This study reveals the potential impact of a hypertension diagnosis on life expectancy (SLE), suggesting a link between hypertension diagnosis and lower SLE, as well as their role in promoting healthier behaviors. Individuals diagnosed with hypertension tend to have lower SLE, even with controlled blood pressure. Providing health education to ease anxiety about hypertension is crucial, as individuals can manage the condition effectively. Our research also highlights proactive behavior among those diagnosed with hypertension, emphasizing the need to enhance their understanding of health risks. Moreover, individuals with measured hypertension often lack awareness and engage in unhealthy behaviors, similar to those diagnosed but with abnormal blood pressure. Enhancing awareness and promoting healthier behaviors are critical for comprehensive health promotion strategies to combat hypertension and its risks.

## Data Availability

The datasets presented in this study can be found in online repositories. The names of the repository/repositories and accession number(s) can be found below: https://charls.pku.edu.cn/en/.
