# Supplementary material for: Influence of hypertension diagnosis and subjective life expectancy on health behaviors among middle-aged and older Chinese adults
Source: Front Public Health. 2025 Jan 6;12:1489284. doi: 10.3389/fpubh.2024.1489284 (PMC11744006; doi:10.3389/fpubh.2024.1489284)
Supplement: Supplementary file 1 [file Data_Sheet_1.docx]

**Influence of Hypertension Awareness and Subjective Life Expectancy on Health Behaviors among Middle-aged and Older Chinese Adults**

SUPPLEMENTARY MATERIAL

**Supplementary Figure S1.** Distribution of subjective life expectancy (SLE)

**Supplementary Table 1.** The difference of health behavior and subjective life expectancy between four groups

| **Dunn's test for hypertensive health behavior score** | | | | |
| --- | --- | --- | --- | --- |
|  | No hypertension | Undiagnosed hypertension | Diagnosed hypertension with normal BP | Diagnosed hypertension with unmoral BP |
| No hypertension | - | 0.275 | -27.440*** | -26.345*** |
| Undiagnosed hypertension | - | - | -22.107*** | -20.941*** |
| Diagnosed hypertension with normal BP | - | - | - | 1.672 |
| Diagnosed hypertension with unmoral BP | - | - | - | - |
| **Dunn's test for general health behavior score** | | | | |
|  | No hypertension | Undiagnosed hypertension | Diagnosed hypertension with normal BP | Diagnosed hypertension with unmoral BP |
| No hypertension | - | 2.012* | -1.675 | -1.847 |
| Undiagnosed hypertension | - | - | -2.894* | -3.048** |
| Diagnosed hypertension with normal BP | - | - | - | -0.082 |
| Diagnosed hypertension with unmoral BP | - | - | - | - |
| **Dunn's test for subjective life expectancy** | | | | |
|  | No hypertension | Undiagnosed hypertension | Diagnosed hypertension with normal BP | Diagnosed hypertension with unmoral BP |
| No hypertension | - | -2.308* | 2.406 | 3.786 |
| Undiagnosed hypertension | - | - | 3.706*** | 4.807*** |
| Diagnosed hypertension with normal BP | - | - | - | 0.991 |
| Diagnosed hypertension with unmoral BP | - | - | - | - |
| *Note: * P<0.05; ** P<0.01; ***P<0.001; N=4864.* | | | | |

**Supplementary Table 2.** Fixed-effects model of diagnosed hypertension on general health behavior

|  | *β*（95% *CI*） |  |
| --- | --- | --- |
| Diagnosed hypertension *(Ref: No d*iagnosed hypertension*)* |  |  |
| Diagnosed hypertension | 0.38 (0.36, 0.41) | *** |
| Age | 0.03 (0.02, 0.03) | *** |
| Gender *(Ref: Male)* |  |  |
| Female | 1.00 (0.97, 1.03) | *** |
| Marital status *(Ref: Married)* |  |  |
| Unmarried | 0.03 (-0.01, 0.07) |  |
| Education *(Ref: Illiterate)* |  |  |
| Elementary school and below | 0.03 (-0.01, 0.07) |  |
| Middle school and above | 0.09 (0.05, 0.12) | *** |
| Residence *(Ref: Urban)* |  |  |
| Rural | -0.08 (-0.12, -0.06) | *** |
| ADLWA | 0.04 (0.01, 0.07) | ** |
| Pension *(Ref: No)* |  |  |
| Yes | -0.23 (-0.27, -0.20) | *** |
| Constant | -0.27 (-0.41, -0.14) | *** |
| N observations | , 5, 223 |  |
| R-squared | , 0.3084226 |  |
| AIC | , 45302.34 |  |
| BIC | , 45388.2758. |  |

*Notes*: CI = Confidence interval; ADLWA = Activities of daily living by Wallace; SLE = Subjective life expectancy; *** p < .001; ** p < .01; * p < .05

**Supplementary Table 3.** Fixed-effects model of SLE on general health behavior

|  | *β*（95% *CI*） |  |
| --- | --- | --- |
| Diagnosed hypertension *(Ref: No d*iagnosed hypertension*)* |  |  |
| Diagnosed hypertension | 0.38 (0.35, 0.41) | *** |
| SLE *(Ref: High-SLE)* |  |  |
| Middle-SLE | 0.06 (0.03, 0.10) | *** |
| Low-SLE | 0.10 (0.07, 0.13) | *** |
| Age | 0.02 (0.02, 0.03) | *** |
| Gender *(Ref: Male)* |  |  |
| Female | 1.00 (0.97, 1.03) | *** |
| Marital status *(Ref: Married)* |  |  |
| Unmarried | 0.03 (-0.01, 0.07) |  |
| Education *(Ref: Illiterate)* |  |  |
| Elementary school and below | 0.03 (-0.01, 0.07) |  |
| Middle school and above | 0.09 (0.04, 0.13) | *** |
| Residence *(Ref: Urban)* |  |  |
| Rural | -0.08 (-0.12, -0.06) | *** |
| ADLWA | 0.04 (0.01, 0.07) | * |
| Pension *(Ref: No)* |  |  |
| Yes | -0.23 (-0.27, -0.19) | *** |
| Constant | -0.31 (-0.45, -0.17) | *** |
| N observations | , 5, 223 |  |
| R-squared | , 0.2893 |  |
| AIC | , 45797.21 |  |
| BIC | , 45898.7758. |  |

*Notes*: CI = Confidence interval; ADLWA = Activities of daily living by Wallace; SLE = Subjective life expectancy; *** p < .001; ** p < .01; * p < .05.

**Supplementary Table 4.** Fixed-effects model of SLE on hypertension-related behavior

|  | *β*（95% *CI*） |  |
| --- | --- | --- |
| SLE *(Ref: High-SLE)* |  |  |
| Middle-SLE | 0.10 (0.04, 0.16) | ** |
| Low-SLE | 0.16 (0.10, 0.22) | *** |
| Age | 0.04 (0.04, 0.04) | *** |
| Gender *(Ref: Male)* |  |  |
| Female | 0.97 (0.92, 1.02) | *** |
| Marital status *(Ref: Married)* |  |  |
| Unmarried | 0.09 (0.02, 0.16) |  |
| Education *(Ref: Illiterate)* |  |  |
| Elementary school and below | 0.02 (-0.04, 0.08) |  |
| Middle school and above | 0.09 (0.02, 0.16) | *** |
| Residence *(Ref: Urban)* |  |  |
| Rural | -0.15 (-0.20, -0.10) | *** |
| ADLWA | 0.04 (0.01, 0.08) | *** |
| Pension *(Ref: No)* |  |  |
| Yes | -0.02 (-0.14, 0.10) |  |
| Constant | -0.63 (-0.87, -0.42) | *** |
| N observations | , 2, 730 |  |
| R-squared | , 0.1283686 |  |
| AIC | , 30971.93 |  |
| BIC | , 31056.068. |  |

*Notes*: CI = Confidence interval; ADLWA = Activities of daily living by Wallace; SLE = Subjective life expectancy; *** p < .001; ** p < .01; * p < .05
